# The nitrogenase mechanism: new roles for the dangler?

**DOI:** 10.1007/s00775-024-02085-7

**Published:** 2024-12-19

**Authors:** Rebeccah A. Warmack, Douglas C. Rees

**Affiliations:** 1 Division of Chemistry and Chemical Engineering, California Institute of Technology, 164-30, Pasadena, CA 91125 USA; 2 Division of Chemistry and Chemical Engineering, Howard Hughes Medical Institute, California Institute of Technology, 147-75, Pasadena, CA 91125 USA

**Keywords:** Nitrogenase, Nitrogen fixation, Iron–sulfur clusters, Metalloproteins

## Abstract

Dangler sites protruding from a core metallocluster were introduced into the bioinorganic lexicon in 2000 by R.D. Britt and co-workers in an analysis of the tetramanganese oxygen-evolving cluster in photosystem II. In this perspective, we consider whether analogous dangler sites could participate in the mechanism of dinitrogen reduction by nitrogenase. Two possible roles for dynamic danglers in the active site FeMo cofactor are highlighted that might occur transiently during turnover. The first role for a dangler involves the S2B belt sulfur associated with displacement by carbon monoxide and other ligands, while the second dangler role could involve the entire cluster upon displacement of the His- $\alpha$ 442 side chain to the molybdenum by a free carboxyl group of the homocitrate ligand. To assess whether waters might be able to interact with the cofactor, a survey of small ligands (water and alkali metal ions) contacting [4Fe4S] clusters in synthetic compounds and proteins was conducted. This survey reveals a preference for these sites to pack over the centers of 2Fe2S rhombs. Waters are excluded from the S2B site in the resting state of nitrogenase, suggesting it is unlikely that water molecules coordinate to the FeMo cofactor during catalysis. While alkali metal ions are found to generally influence the properties of catalysts for dinitrogen reduction, no convincing evidence was found that any of the waters near the FeMo cofactor could instead be sodium or potassium ions. Dangler sites, if they exist in the nitrogenase mechanism, are likely formed transiently by localized changes to the resting-state FeMo cofactor structure.

**Graphical abstract:**

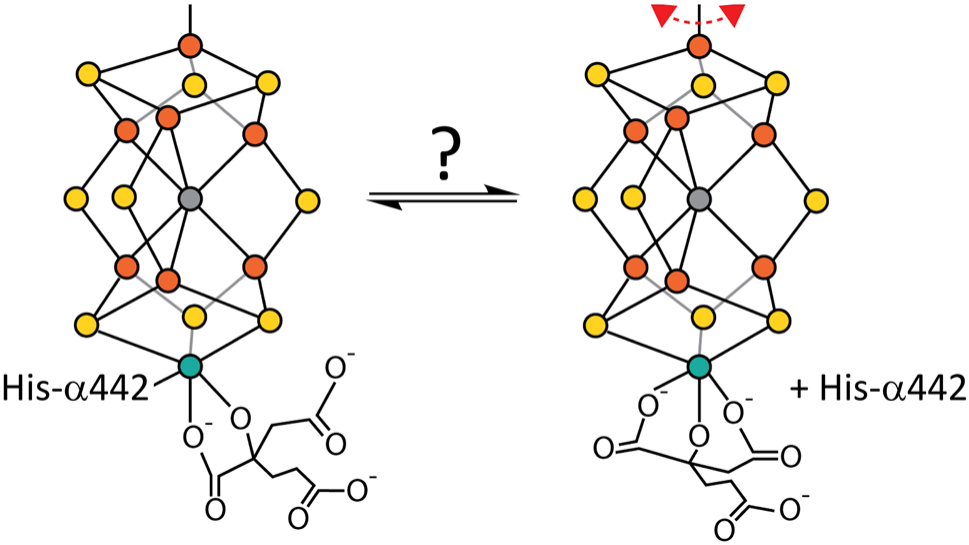

## Introduction

This celebration of R. David Britt not only recognizes his selection to receive the Alfred Bader Award in Bioinorganic or Bioorganic Chemistry, but more generally serves as a richly fitting tribute to a remarkable scientist and person. One of the authors (DCR) has had the great pleasure of interacting with Dave since the 1980s, initially through meetings reflecting shared interests in photosynthesis and the applications of synchrotron radiation to the study of complex metalloproteins. Dave’s work is characterized by his rigorous science, complete integrity, and total absence of “BS”. He understands and speaks many “languages”—the quantum mechanics of spins, spectroscopy, biochemistry, enzymology, synthetic chemistry—that are clearly reflected in his own research and his extensive network of collaborators. Dave is a tremendous ambassador for chemistry and science generally, and makes science fun, which is no small accomplishment these days when there is so much stress associated with the actual practice of science.

This contribution was motivated by Dave’s beautiful work on the mechanism of water oxidation by photosystem II. Water oxidation occurs at the oxygen-evolving complex, an Mn_4_Ca-containing metallocluster, which has been a focus of extensive studies over the decades aimed at deciphering the catalytic mechanism (a historical review may be found in [1]). Over 20 years ago, Dave’s group made the seminal observation using pulsed electron paramagnetic resonance approaches (i.e., sporting methods) that one of the manganese (Mn) sites in the oxygen-evolving complex was weakly coupled to the cluster core containing the other three Mn [2, 3]. Dave described this as the “dangler model” and subsequent studies have established the dangler Mn plays a key role in the water oxidation reaction that yields dioxygen. Dave’s group has more recently established a role for a dangler Fe in the enzyme generating an organometallic precursor to the [FeFe]-hydrogenase H cluster [4].

In this perspective, we describe speculative roles for dangler sites in the mechanism of nitrogenase, the enzyme catalyzing the adenosine triphosphate-dependent reduction of dinitrogen to ammonia. Nitrogenase has interesting parallels to photosystem II; both enzymes perform reactions that couple cellular metabolism to critical environmental inputs (water and solar radiation for photosystem II, and dinitrogen for nitrogenase). These catalytic mechanisms involve a sequence of electron/proton transfers mediated by complex metalloclusters that have been attractive targets for biophysical studies, particularly electron paramagnetic resonance, X-ray absorption spectroscopy and crystallography; the latter studies were made possible by the development of X-ray synchrotron sources that provided the background for DCR’s early interactions with Dave. In other ways, though, nitrogenase and photosystem II are more like polar opposites. Photosystem II catalyzes the oxidation of water to yield dioxygen at a high potential cluster, while nitrogenase catalyzes the reduction of dinitrogen to ammonia at a low potential, oxygen-sensitive cluster. Light has great advantages as a “substrate” to sequentially drive photosystem II through successive steps in the catalytic cycle, and as a consequence, the mechanistic understanding of photosystem II is advanced relative to that of nitrogenase. The dangler Mn plays a key role in the mechanism of oxygen evolution, and here we consider whether there could be analogous sites in the nitrogenase mechanism.

## Danglers in the nitrogenase mechanism?

**Fig. 1:**
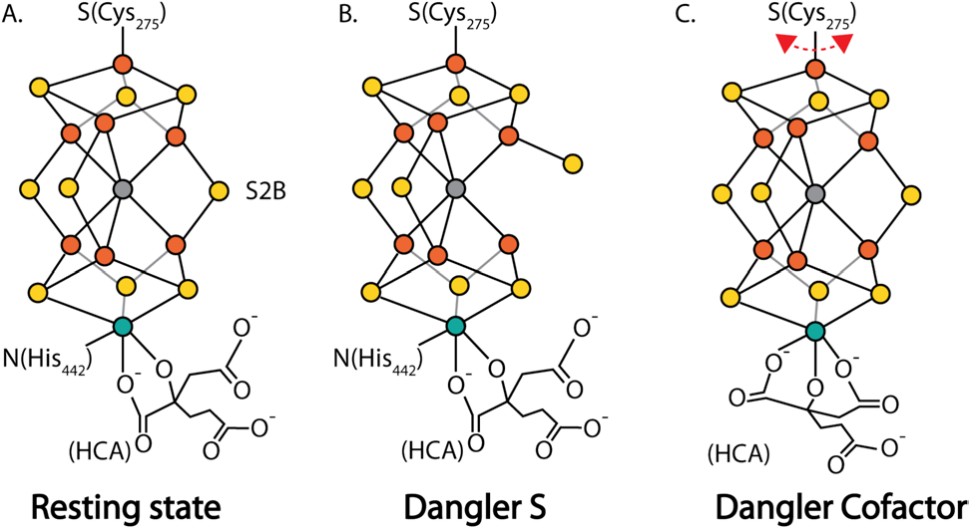
Schematic representations of the nitrogenase FeMo cofactor in the **A** resting state, and in two hypothetical dynamic dangler forms that could be generated under turnover conditions: **B** with a dangling belt sulfur generating a reactive Fe site in the central Fe_6_C trigonal prism and **C** by displacement of the His- $\alpha$ 442 ligand by a free homocitrate carboxyl group to generate a dangling cofactor species coordinated to the protein through only one ligand. The existence of this species during turnover could provide a mechanism for the observed interchange of Se among the belt sulfur positions [15]; this state could also participate in non-turnover processes, such as cofactor insertion and repair of damaged cofactor

As this perspective is focused on the active site of nitrogenase, the reader is invited to peruse recent reviews that provide a more complete overview of the nitrogenase system [5–9]. The active site of nitrogenase is provided by the iron–molybdenum (FeMo-) cofactor, together with the surrounding protein residues. The FeMo cofactor is a [7Fe:9S:1Mo:1C]-*R*-homocitrate metallocluster with a remarkable structure (Fig. 1A); the core of the cofactor is formed by a trigonal prismatic core of six Fe surrounding an interstitial carbon, with three pairs of “belt sulfurs” bridging between pairs of Fe in the two triangular faces. The FeMo cofactor is coordinated to the protein through the sidechains of two residues, Cys- $\alpha$ 275 and His- $\alpha$ 442, that bind the terminal Fe and Mo, respectively. Significantly, no protein ligands coordinate any of the six Fe forming the trigonal prism. The coordination sphere of the Mo is completed through bidentate coordination by homocitrate, a hydroxytricarboxylic acid that possesses an extra methylene group in one “arm” relative to citrate. Homocitrate coordination to molybdenum utilizes the hydroxyl group and adjacent carboxylate group (Fig. 1A); the two terminal carboxylate groups do not participate in metal coordination in the resting state. Alternate nitrogenases exist with related cofactor structures, differing notably in the replacement of Mo by V or Fe, but still retaining the homocitrate [10, 11]; this discussion will focus on the FeMo cofactor. As is evident from Fig. 1A, there are no danglers, i.e., sites protruding from the FeMo cofactor, in the resting state.

It is important to recognize that the FeMo cofactor is a cofactor, which means it can be extracted from the mature protein and then used to reconstitute a cofactor deficient form of nitrogenase [12]. This property requires that the resting state of the FeMo cofactor must be sufficiently stable to survive outside of the protein. However, during the catalytic cycle, the FeMo cofactor must become sufficiently reactive to bind and reduce the kinetically inert N_2_. Reconciling these two extremes, stability of the resting state and high reactivity during the catalytic cycle, requires that not only the substrate but also the cofactor be transiently activated during the reaction cycle [13]. One clue as to how this activation step might happen was provided by the crystallographic characterization of a stably trapped carbon monoxide (CO)-containing species generated under turnover conditions [14]. The CO was observed to displace the belt sulfur site designated S2B, bridging two Fe along one edge of the central trigonal prism. A series of subsequent structural studies have highlighted the ability of this site to accommodate a variety of ligands ranging from one CO, two COs, Se, and light atom (O/N) species potentially arising under turnover conditions [15–20]. The conserved nature of the Fe_6_C core [17, 21], together with the demonstrated ability of this site to bind exogenous ligands, has focused attention on this region as the site for substrate binding and activation.

How can the S2B site open to bind exogenous ligands under turnover conditions? Computational studies have generally found that the belt sulfurs, and S2B in particular, are predicted to be the most basic sites on the cofactor [22–24]. Protonation of the S2B site in more highly reduced forms of the cofactor generated during turnover should enhance dissociation of this ligand from one of the Fe, thereby generating a “dynamic dangler”, i.e., a transiently protruding S species (Fig. 1B). In effect, S2B would serve as a protecting group that blocks substrate binding to the cofactor until the appropriate state when it is deprotected [14]. Computational studies have identified potential mechanisms initially involving dissociation of S2B from one Fe, yielding a terminal HS^−^ ligand (see [24–26]). This dangler S (Fig. 1B) could play an essential role in the catalytic mechanism by generating a transient site in the trigonal prism core for substrate binding and consequent reduction. It is also possible that a dangler S could serve as an intermediate on a pathway to complete dissociation of S2B or potentially another cofactor S (see, for example, [27]).

The selective incorporation of exogenous compounds into the FeMo cofactor under turnover conditions provided an experimental approach to probe for metallocluster rearrangements during substrate reduction. Nitrogenase catalyzes the incorporation of Se from selenocyanate (SeCN^−^) predominantly into the S2B position of the FeMo cofactor [15]. Starting with this site-specifically labeled sample, a spatially analyzed “pulse-chase” experiment was conducted by incubating nitrogenase initially labeled with Se at the S2B site under turnover conditions in the presence of the reducible substrate acetylene, but not SeCN^−^. The reaction mixture was sampled at multiple time points, crystallized, and the distribution of selenium crystallographically determined using diffraction data collected at the selenium absorption edge. Remarkably, the Se initially at the S2B position was found to migrate to the other two belt sulfur positions, and with enough turnovers, the selenium was eventually “chased” from the cofactor. This Se-labeling experiment established that the belt sulfur positions are scrambled during turnover.

How does this scrambling occur? One possibility is that there is an internal rearrangement that interconverts the belt sulfur positions. Dance explored potential mechanisms computationally and found that there would be a high energetic barrier that would seem to preclude this type of scrambling process [28]. An alternative mechanism was suggested by our recent analysis [29] of the turnover-dependent inactivation of nitrogenase under high pH conditions [30]. This work revealed the possibility of altered coordination of Mo by homocitrate and His- $\alpha$ 442 during turnover. A driving force for this transformation could be the tendency for homocitrate, citrate, and related molecules to form tridentate metal complexes utilizing the hydroxy group and two carboxylates as metal ligands [31]. In the case of nitrogenase, we speculate that binding of one of the initially unliganded terminal carboxylates to Mo might displace the His- $\alpha$ 442 side chain, resulting in tridentate coordination of the Mo by homocitrate (Fig. 1C). As a consequence, the FeMo cofactor would only be tethered to the protein through the single Fe-Cys- $\alpha$ 275 linkage. In essence, the entire cofactor would be “dangling” from the protein. Rotation of the entire FeMo cofactor about the Fe-Cys- $\alpha$ 275 linkage would thereby interconvert the belt sulfur positions, which could account for the scrambling observed in the Se-labeling experiment [29]. We note that a “rotating sites” model has been proposed by Ribbe and Hu [32, 33], with the substrate shifting between sites on the cofactor, rather than the entire cofactor rotating. A dangling cofactor, free to rotate in the active site, provides a simple mechanism for changing the environment of the cofactor during the catalytic cycle.

Changes in the homocitrate coordination of molybdenum have previously been proposed, specifically the loss of one of the two ligating interactions, as a mechanism to generate a binding site for substrates on Mo [34, 35]. To our knowledge, the possibility that homocitrate may become a tridentate ligand to Mo during turnover has not been proposed (although it would be a plausible species to “cap” the Mo during insertion of the FeMo cofactor into the apo-MoFe-protein during assembly). Whether a second homocitrate carboxylate group can indeed displace His- $\alpha$ 442 as a Mo ligand is completely speculative at this point, but we note that a precedent for the ability of a citrate-type ligand to exhibit multiple coordination modes to an FeS cluster is provided by the enzyme aconitase. Aconitase catalyzes the isomerization of citrate to isocitrate in one of the steps of the citric acid cycle. The aconitase mechanism is conventionally described as involving the initial coordination of citrate to a single Fe of a [4Fe4S] cluster through the central hydroxyl and carboxylate groups [36]. Following a base-catalyzed, stereospecific deprotonation of one of the citrate methylene groups, the hydroxyl group of citrate is lost as water, yielding *cis-*aconitate. *cis*-Aconitate is then proposed to “flip” in the active site through a change in the carboxylate group coordinating the cluster. Following stereospecific hydration to yield isocitrate, the product dissociates. Intriguingly, the methylene carbon in the short “arm” of homocitrate is 3.7 Å from the S3B cluster sulfur of the FeMo cofactor, a potentially basic site. Two diastereomers, *threo*- and *erythro*-, are generated when one of the hydrogens of this methylene is replaced with fluorine; the *threo*- and *erythro*- substituents points toward and away from S3B, respectively. Ludden [37, 38] has reported the intriguing observation that the *erythro*-diastereomer can reduce dinitrogen, while the *threo*-diastereomer cannot (the two diastereomers reduce acetylene and protons at comparable rates). Perhaps this is a steric effect, but it might also indicate that this methylene proton of homocitrate is chemically important for the dinitrogen reduction. The relative acidity of these methylene protons might also be relevant to the turnover-dependent inactivation of nitrogenase at high pH [30].

## FeS clusters, ions and hydrophobia

In addition to dynamic danglers resulting from internal rearrangements of the FeMo cofactor during turnover, other possible mechanisms to generate protruding dangler sites could involve addition of exogenous species, such as waters from the surrounding environment. Water plays an essential role in the nitrogenase mechanism as the ultimate source of the significant proton requirement of 8 (or 10) protons for the pair of NH_3_ (or NH_4_^+^) and the obligatory H_2_ produced per N_2_ reduced. Waters have been proposed to participate in proton transfer pathways that have been identified based on the resting-state structure [39–41]. Here, we rely on the numbering of waters in the 3U7Q PDB coordinate set [42]. In that structure, several well-ordered water molecules are bound near the cofactor on the side of the S5A belt sulfur [43]: water A519 near the Fe7-Mo1 rhomb (3.8 Å from S4B, and hydrogen bonded to homocitrate), and waters A529, A537, and A542 near the Fe1-Fe3 rhomb (~ 4.2 Å from S4A). Interestingly, no water molecules in the resting-state structure are present near the belt sulfur S2B site associated with ligand-binding.

As a starting point for assessing how water might interact with the FeMo cofactor, we have examined this interaction in FeS clusters from both synthetic systems and metalloproteins. For the synthetic clusters, the Cambridge Structural Database [44] was searched using WebCSD for compounds containing 4Fe and 8S in the molecular formula, and then manually searching the 155 output records for structures with waters near a [4Fe4S] cluster. Only one such interaction was found (CSD refcode 114,011, [45]), a reflection that most such compounds have been synthesized with hydrophobic ligands and counterions (typically bulky quaternary amines) and are crystallized in non-aqueous solvents (the source of the water in 114,011 was thought to be “wet” ethyl acetate). Indeed, even when thiol ligands with carboxylate groups are used, the crystal structure of a water soluble [4Fe4S] cluster revealed hydrophobic quaternary amines packed against the sides of the cluster [46]. This overall behavior indicates that FeS clusters have an apolar character.

**Fig. 2:**
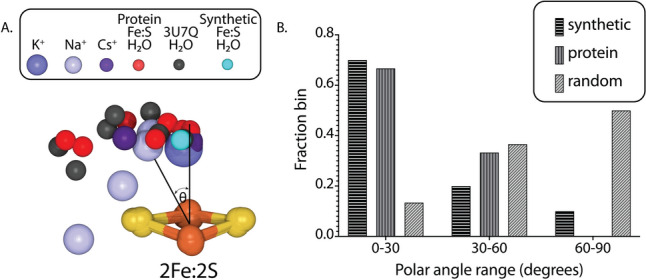
Distribution of alkali metals and waters about a 2Fe2S rhomb observed in structures of synthetic clusters and cluster containing proteins. **A** Superposition of binding sites relative to a reference 2Fe2S rhomb, oriented such that the closest interaction of the ligand is to the left-most S, and the ligand is above the plane of the 2Fe2S rhomb. Ligands Na^+^, K^+^, Cs^+^, and water are from the structures of synthetic clusters. The red spheres are water molecules from protein structures containing [4Fe4S] clusters solved at resolutions < 1.1 Å, while the dark gray spheres represent the positioning of waters near the nitrogenase metalloclusters (PDB 3U7Q), and the cyan sphere corresponds to only water observed to interact with a synthetic FeS cluster. The polar angle θ is defined as the angle between the normal to the 2Fe2S rhomb and the vector from the rhomb centroid to the ligand. **B** Histogram of the binned distribution of the polar angle θ for ligands in synthetic compounds and FeS proteins, along with the expected values for a random distribution. A strong preference is observed for these ligands to be oriented above the centroid of the 2Fe2S rhomb, in both small molecules and in protein structures

While waters are rarely found adjacent to synthetic FeS clusters, a larger set of synthetic [4Fe4S] clusters has been found with alkali metals as counterions in proximity to the cluster. Structurally characterized systems include Na^+^ (CSD 1287654 [47, 48]), K^+^ (CSD 2124635, 2,124,638 [49]), and Cs^+^ (CSD 1597303, 2,244,603 [50, 51]). These cations were found to have interactions with a sulfur on one of the 2Fe2S rhombs, with the closest distances to this S for Na^+^, K^+^ and Cs^+^ averaging 3.02 Å ± 0.13 Å (n = 4), 3.19 Å ± 0.01 Å (n = 2), and 3.57 Å ± 0.02 Å (n = 3), respectively. For reference, the water in CSD 11401 is 4.10 Å from the nearest sulfur. A graphical representation of these interactions was generated by superposing the associated 2Fe2S rhombs, with the Fe–Fe vector defining the x-axis, the S of closest contact oriented along y, and the alkali metal/water site having positive z (Fig. 2A).

As an initial assessment of waters interacting with protein bound FeS clusters, we searched the Research Collaboratory for Structural Bioinformatics Protein Data Bank (PDB; [52]) for structures with [4Fe4S] clusters (PDB group SF4) solved at resolutions better than 1.1 Å. Of the 30 structures satisfying this criteria, 11 structures are unique, and these structures contain 19 [4Fe4S] clusters. While hydrogen bonds from protein residues to cluster sulfides [53] were present in all structures, only 9 close interactions between water and [4Fe4S] clusters were identified in 4 of these structures (PDB IDs 1IQZ, 4UDX, 4U9H and 6N59). The distances from these waters to the nearest cluster S average 3.7 Å ± 0.3 Å, longer than observed for either Na^+^ or K^+^ ions. The positions of these waters relative to the nearest 2Fe2S rhomb in these structures are illustrated in Fig. 2A, using the same convention as for the synthetic clusters. For reference, the 7 water molecules in the nitrogenase MoFe-protein 3U7Q coordinate set within 4.2 Å of either the FeMo cofactor (A519, A529, A537, and A542) or the P-cluster (B527, B541, and B576) are also depicted.

The distributions of water and ions around a 2Fe2S rhomb appear non-random, with an apparent preference for binding sites positioned over the center of the ring, in a manner reminiscent of cation– π interactions near aromatic residues in proteins [54]. The location of sites relative to the centroid of the rhomb can be represented by the polar angle θ, where θ is the angle between the normal to the rhomb and the vector from the centroid to the water/ion site (Fig. 2A). The random probability distribution of θ, P(θ) = sin(θ), appears in a variety of protein structure contexts, including the packing of aromatic sidechains in proteins [55]. For comparing the observed and random distributions, the polar angle distribution was calculated in 30˚ bins given the small observed sample size (Fig. 2B). A comparison of the polar angle distribution of water/ions around FeS clusters relative to the expected random distribution confirms that there is a significant preference for packing over the face of the 2Fe2S rhombs. While water/ions are also observed in the rhomb plane, the frequency is less than what would be expected for a random distribution.

## Dangling waters or dangling ions?

The observation that cations can interact with FeS clusters raises the important question—are all the waters really water in the nitrogenase structure or could some of them actually be ions? This distinction is important in nitrogenase not only to have the most accurate structural model, but also since alkali metals have been implicated as conferring favorable properties in nitrogen fixation catalysts [56]. It can be difficult to distinguish ions from water in electron density maps by X-ray crystallography, especially Na^+^ or K^+^ with partial occupancy. Typically, these assignments are indirectly inferred from the coordination number and the distances to surrounding groups. The combination of electron microscopy (EM) and X-ray crystallography can, however, help resolve ambiguous cases since electron scattering is impacted by the charge of the scatterer. Specifically, positively charged groups look stronger (and negative charges look weaker) than neutral species, and the effect is most significant at low resolution (see the discussion in [57]). A comparison of B factors for X-ray and EM structures provides an approach to identify solvent sites that are potentially ions.

**Fig. 3:**
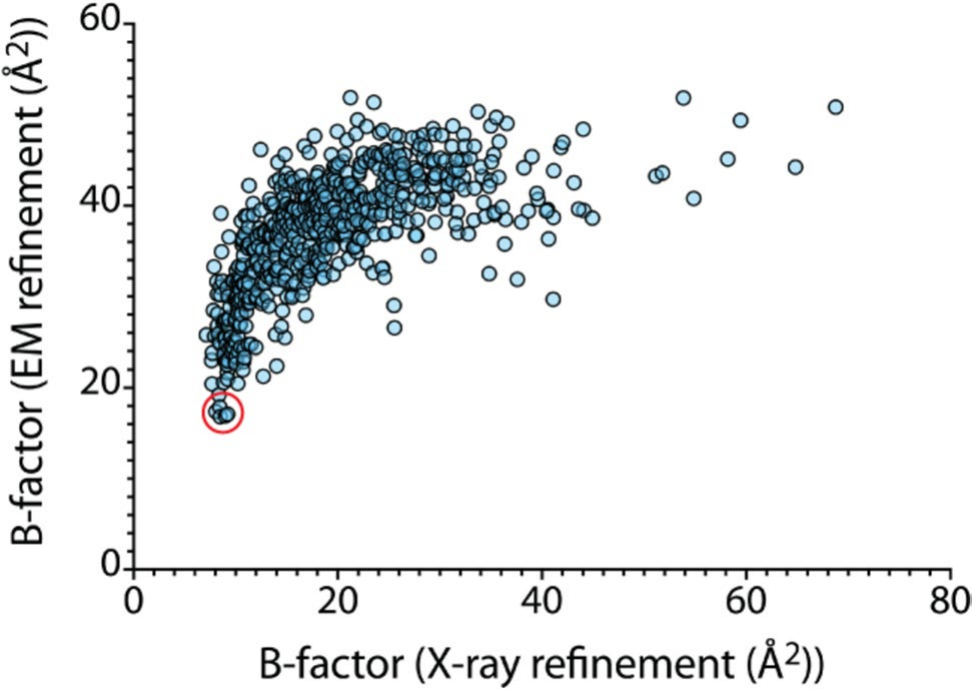
Correlation of B factors observed for solvent molecules in the X-ray and EM refinements of the *A. vinelandii* MoFe-protein (PDB IDs 3U7Q and 8CRS, respectively). The red circle highlights water molecules with the lowest B factors in the EM refinement that could potentially represent cations

To assess potential cation-binding sites in nitrogenase, we generated a list of solvent sites present in both the X-ray and EM maps of the MoFe-protein (PDBs 3U7Q and 8CRS, respectively). 610 unique solvent molecules were selected for this analysis that reflect the two-fold molecular symmetry of nitrogenase to within 1 Å (this corresponds to 1220 waters for the entire MoFe-protein tetramer; for reference, 2602 and 1692 waters are present in 3U7Q and 8CRS, respectively). The average values of temperature factors for the twofold-related waters were calculated separately for the X-ray and EM structures and then plotted against the other (Fig. 3).

Several observations:

- While the two sets of Bs are correlated, the relationship is non-linear so that the refinements of one or both of the X-ray and EM structures must be impacted by systematic effects whose origins are unclear from this study.
- Six solvents have B factors in the EM refinement below ~ 20 Å^2^ (red circle in Fig. 3). While these solvent molecules also have low X-ray B factors, they are not uniquely low, and hence these could potentially correspond to cations based on the criteria that they have stronger density in the EM map relative to the X-ray map as reflected by lower B values. Intriguingly, among these six sites are two conserved sites near the FeMo cofactor and P-cluster (A519 and B527, respectively).
- Could water A519 actually be a cation? On closer examination, this possibility seems unlikely. The expected metal–water distances for Na^+^ and K^+^ are 2.42 Å and 2.84 Å, respectively [58]. The distances from A519 to the 3 surrounding oxygen ligands average ~ 3.0 Å which rules out Na^+^ used in the purification and crystallization solutions. While these distances match those expected for K^+^ (the dominant intracellular cation), a full occupancy K^+^ ion would be expected to have a significantly lower (~ 50%) B factor than an equivalent water [59], which is not the case for this site. While a partial occupancy for K^+^ cannot be ruled out, this interpretation seems less likely considering how well this site is conserved.

## Dangling into thin air?

Nitrogenase has been described as the Everest of Enzymes for the “daunting number of unanswered questions about the mechanism of nitrogenase activity” [60]. We conclude this perspective by paraphrasing W.N. Lipscomb [61] and venturing “a few predictions, knowing that if we must join the ranks of (nitrogenase mechanism) predictors later proved wrong, we shall be in the best of company”.

- Are there danglers in the nitrogenase mechanism? yes! It seems likely to us that S2B becomes disconnected from at least one Fe, creating a dynamic dangler site. Moving further out on a limb, we suggest that one role of the homocitrate is to form a tridentate complex with the Mo of the FeMo cofactor by displacing His-$\alpha$ 442 and thereby generating a dangling cofactor that can rotate around the Fe1–Cys-$\alpha$275 bond. This dangler could not only be involved in insertion of the cofactor during nitrogenase biosynthesis, but also during turnover and, we propose [29], as part of a repair pathway by which damaged cofactor can be exchanged for new cofactor.
- Are there any dangling species generated by addition of water to the FeMo cofactor? No! While there are waters near the cofactor, there are none present in the resting state near the putative ligand-binding site (S2B). An earlier computational studies proposed that water could attack an Fe during the nitrogenase mechanism [62], but this feature does not seem present in more recent computational models. We note that the extracted cofactor is unstable in an aqueous environment [12, 63] and that [4Fe4S] clusters are sensitive to acid-catalyzed solvolysis [64, 65]. Consequently, exclusion of water from the S2B region could reflect an adaptation to prevent reactivity with water, thereby avoiding formation of a “dead dangler” species.
- Do alkali ions participate in the nitrogenase mechanism? Probably not, as there is no compelling reason to believe any of the solvent molecules near the cofactor could be Na^+^ or K^+^ (at least in the resting state). We note that we are referring to a specific catalytic role for alkali metal ions, which is quite distinct from the effects of ionic strength on the nitrogenase reaction [66, 67].

From this speculative reasoning, we conclude that dangler sites, if they exist in the nitrogenase mechanism, are likely formed transiently by localized changes to the resting-state FeMo cofactor structure.

## Data Availability

No datasets were generated or analysed during the current study.

## Abbreviations

*CO*: Carbon monoxide
CSD: Cambridge Structural Data Base
*Cys*: Cysteine
*EM*: Electron microscopy
*FeMo cofactor*: Iron–molybdenum cofactor
*FeS*: Iron–sulfur
*His*: Histidine
*MoFe-protein*: Nitrogenase molybdenum-iron protein
PDB: Protein Data Bank
*SeCN*^−^: Selenocyanate ion
*2Fe2S*: The face of an FeS cluster with 2 Fe and 2 S.
*4Fe4S*: An iron–sulfur cluster with four irons and four sulfurs

## References

[1] Junge W (2019) Oxygenic photosynthesis: history, status and perspective. Quart Rev of Biophy. 10.1017/s0033583518000112 10.1017/S0033583518000112 30670110

[2] Peloquin JM, Britt RD (2001) EPR/ENDOR characterization of the physical and electronic structure of the OEC Mn cluster. BBA-Bioenergetics 1503:96–111. 10.1016/s0005-2728(00)00219-x 11115627 10.1016/s0005-2728(00)00219-x

[3] Peloquin JM, Campbell KA, Randall DW, Evanchik MA, Pecoraro VL, Armstrong WH, Britt RD (2000) 55Mn ENDOR of the S2-state multiline EPR signal of photosystem II: Implications on the structure of the tetranuclear Mn cluster. J Am Chem Soc 122:10926–10942. 10.1021/ja002104f

[4] Suess DLM, Pham CC, Bürstel I, Swartz JR, Cramer SP, Britt RD (2016) The radical SAM enzyme HydG requires cysteine and a dangler iron for generating an organometallic precursor to the FeFe-hydrogenase H-cluster. J Am Chem Soc 138:1146–1149. 10.1021/jacs.5b12512 26764535 10.1021/jacs.5b12512 PMC4772725

[5] Warmack RA, Rees DC (2023) Nitrogenase beyond the resting state: a structural perspective. Molecules. 10.3390/molecules28247952 38138444 10.3390/molecules28247952 PMC10745740

[6] Rutledge HL, Tezcan FA (2020) Electron transfer in nitrogenase. Chem Rev 120:5158–5193. 10.1021/acs.chemrev.9b00663 31999100 10.1021/acs.chemrev.9b00663 PMC7466952

[7] Seefeldt LC, Yang ZY, Lukoyanov DA, Harris DF, Dean DR, Raugei S, Hoffman BM (2020) Reduction of substrates by nitrogenases. Chem Rev 120:5082–5106. 10.1021/acs.chemrev.9b00556 32176472 10.1021/acs.chemrev.9b00556 PMC7703680

[8] Einsle O, Rees DC (2020) Structural enzymology of nitrogenase enzymes. Chem Rev 120:4969–5004. 10.1021/acs.chemrev.0c00067 32538623 10.1021/acs.chemrev.0c00067 PMC8606229

[9] Gu WY, Milton RD (2020) Natural and engineered electron transfer of nitrogenase. Chem-Switzerland. 10.3390/chemistry2020021

[10] Burén S, Jiménez-Vicente E, Echavarri-Erasun C, Rubio LM (2020) Biosynthesis of nitrogenase cofactors. Chem Rev 120:4921–4968. 10.1021/acs.chemrev.9b00489 31975585 10.1021/acs.chemrev.9b00489 PMC7318056

[11] Jasniewski AJ, Lee CC, Ribbe MW, Hu YL (2020) Reactivity, mechanism, and assembly of the alternative nitrogenases. Chem Rev 120:5107–5157. 10.1021/acs.chemrev.9b00704 32129988 10.1021/acs.chemrev.9b00704 PMC7491575

[12] Shah VK, Brill WJ (1977) Isolation of an iron-molybdenum cofactor from nitrogenase. Proc Natl Acad Sci USA 74:3249–3253 410019 10.1073/pnas.74.8.3249 PMC431518

[13] Buscagan TM, Rees DC (2019) Rethinking the nitrogenase mechanism: activating the active site. Joule 3:2662–2678. 10.1016/j.joule.2019.09.004 32864580 10.1016/j.joule.2019.09.004 PMC7451245

[14] Spatzal T, Perez KA, Einsle O, Howard JB, Rees DC (2014) Ligand binding to the FeMo-cofactor: structures of CO-bound and reactivated nitrogenase. Science 345:1620–1623. 10.1126/science.1256679 25258081 10.1126/science.1256679 PMC4205161

[15] Spatzal T, Perez KA, Howard JB, Rees DC (2015) Catalysis-dependent selenium incorporation and migration in the nitrogenase active site iron-molybdenum cofactor. Elife. 10.7554/eLife.11620 26673079 10.7554/eLife.11620 PMC4755756

[16] Buscagan TM, Perez KA, Maggiolo AO, Rees DC, Spatzal T (2021) Structural characterization of two CO molecules bound to the nitrogenase active site. Angewandte Chemie-Int Edition 60:5704–5707. 10.1002/anie.202015751 10.1002/anie.202015751 PMC7920927 33320413

[17] Trncik C, Detemple F, Einsle O (2023) Iron-only Fe-nitrogenase underscores common catalytic principles in biological nitrogen fixation. Nat Catal. 10.1038/s41929-023-00952-1

[18] Rohde M, Laun K, Zebger I, Stripp ST, Einsle O (2021) Two ligand-binding sites in CO-reducing V nitrogenase reveal a general mechanistic principle. Sci Adv. 10.1126/sciadv.abg4474 34049880 10.1126/sciadv.abg4474 PMC8163085

[19] Rohde M, Grunau K, Einsle O (2020) CO binding to the FeV cofactor of CO-reducing vanadium nitrogenase at atomic resolution. Angewandte Chemie-Int Edition 59:23626–23630. 10.1002/anie.202010790 10.1002/anie.202010790 PMC7756900 32915491

[20] Sippel D, Rohde M, Netzer J, Trncik C, Gies J, Grunau K, Djurdjevic I, Decamps L, Andrade SLA, Einsle O (2018) A bound reaction intermediate sheds light on the mechanism of nitrogenase. Science. 10.1126/science.aar2765 29599235 10.1126/science.aar2765

[21] Decamps L, Rice DB, DeBeer S (2022) An Fe_6_C core in all nitrogenase cofactors. Angewandte Chemie-Int Edition. 10.1002/anie.202209190 10.1002/anie.202209190 PMC9826452 35975943

[22] Cao LL, Caldararu O, Ryde U (2018) Protonation and reduction of the FeMo cluster in nitrogenase studied by quantum mechanics/molecular mechanics (QM/MM) calculations. J Chem Theory Comput 14:6653–6678. 10.1021/acs.jctc.8b00778 30354152 10.1021/acs.jctc.8b00778

[23] Van Stappen C, Thorhallsson AT, Decamps L, Bjornsson R, DeBeer S (2019) Resolving the structure of the E_1_ state of Mo nitrogenase through Mo and Fe K-edge EXAFS and QM/MM calculations. Chem Sci 10:9807–9821. 10.1039/c9sc02187f 32055350 10.1039/c9sc02187f PMC6984330

[24] Dance I (2022) Understanding the tethered unhooking and rehooking of S2B in the reaction domain of FeMo-co, the active site of nitrogenase. Dalton Trans 51:15538–15554. 10.1039/d2dt02571j 36168836 10.1039/d2dt02571j

[25] Jiang H, Svensson OKG, Ryde U (2022) QM/MM study of partial dissociation of S2B for the E2 intermediate of nitrogenase. Inorg Chem 61:18067–18076. 10.1021/acs.inorgchem.2c02488 36306385 10.1021/acs.inorgchem.2c02488 PMC9667496

[26] Pang YJ, Bjornsson R (2023) Understanding the electronic structure basis for N_2_ binding to FeMoco: a systematic quantum mechanics/molecular mechanics investigation. Inorg Chem 62:5357–5375. 10.1021/acs.inorgchem.2c03967 36988551 10.1021/acs.inorgchem.2c03967 PMC10091479

[27] Wei WJ, Siegbahn PEM (2022) A mechanism for nitrogenase including loss of a sulfide. Chem-a Europ J. 10.1002/chem.202103745 10.1002/chem.202103745 PMC9303661 35098591

[28] Dance I (2016) Mechanisms of the S/CO/Se interchange reactions at FeMo-co, the active site cluster of nitrogenase. Dalton Trans 45:14285–14300. 10.1039/c6dt03159e 27534727 10.1039/c6dt03159e

[29] Warmack RA, Maggiolo AO, Orta A, Wenke BB, Howard JB, Rees DC (2023) Structural consequences of turnover-induced homocitrate loss in nitrogenase. Nat Commun. 10.1038/s41467-023-36636-4 36841829 10.1038/s41467-023-36636-4 PMC9968304

[30] Yang KY, Haynes CA, Spatzal T, Rees DC, Howard JB (2014) Turnover-dependent inactivation of the nitrogenase MoFe-protein at high pH. Biochemistry 53:333–343. 10.1021/Bi4014769 24392967 10.1021/bi4014769 PMC3932303

[31] Wright DW, Chang RT, Mandal SK, Armstrong WH, OrmeJohnson WH (1996) Novel vanadium(V) homocitrate complex: synthesis, structure, and biological relevance of [K_2_(H_2_O)_5_][(VO_2_)_2_(*R, *S-homocitrate_)_2] • _H_2*O*. J Biol Inorg Chem 1:143–151. 10.1007/s007750050033

[32] Ribbe MW, Hu YL (2023) Belt-sulfur mobilization in nitrogenase biosynthesis and catalysis. Trends in Chemistry 5:108–111. 10.1016/j.trechm.2022.12.001 38463155 10.1016/j.trechm.2022.12.001 PMC10923593

[33] Kang W, Lee CC, Jasniewski AJ, Ribbe MW, Hu YL (2020) Structural evidence for a dynamic metallocofactor during N_2_ reduction by Mo-nitrogenase. Science. 10.1126/science.aaz6748 32554596 10.1126/science.aaz6748 PMC8410457

[34] Pickett CJ (1996) The Chatt cycle and the mechanism of enzymic reduction of molecular nitrogen. J Biol Inorg Chem 1:601–606

[35] Gronberg KLC, Gormal CA, Durrant MC, Smith BE, Henderson RA (1998) Why R-Homocitrate is essential to the reactivity of FeMo-cofactor of nitrogenase: Studies on NifV–extracted FeMo-cofactor. J Am Chem Soc 120:10613–10621

[36] Beinert H, Kennedy MC, Stout CD (1996) Aconitase as iron-sulfur protein, enzyme, and iron-regulatory protein. Chem Rev 96:2335–2373. 10.1021/cr950040z 11848830 10.1021/cr950040z

[37] Madden MS, Kindon ND, Ludden PW, Shah VK (1990) Diastereomer-dependent substrate reduction properties of a dinitrogenase containing 1-fluorohomocitrate in the iron-molybdenum cofactor. Proc Natl Acad Sci USA 87:6517–6521. 10.1073/pnas.87.17.6517 2204057 10.1073/pnas.87.17.6517 PMC54567

[38] Ludden PW, Shah VK, Roberts GP, Homer M, Allen R, Paustian T, Roll J, Chatterjee R, Madden M, Allen J (1993) Biosynthesis of the iron molybdenum cofactor of nitrogenase. ACS Symp Ser 535:196–215

[39] Durrant MC (2001) Controlled protonation of iron-molybdenum cofactor by nitrogenase: a structural and theoretical analysis. Biochemical Journal 355:569–576 11311117 10.1042/bj3550569 PMC1221770

[40] Dance I (2012) The controlled relay of multiple protons required at the active site of nitrogenase. Dalton Trans 41:7647–7659. 10.1039/c2dt30518f 22609731 10.1039/c2dt30518f

[41] Dance I (2015) The pathway for serial proton supply to the active site of nitrogenase: enhanced density functional modeling of the Grotthuss mechanism. Dalton Trans 44:18167–18186. 10.1039/c5dt03223g 26419970 10.1039/c5dt03223g

[42] Spatzal T, Aksoyoğlu M, Zhang LM, Andrade SLA, Schleicher E, Weber S, Rees DC, Einsle O (2011) Evidence for interstitial carbon in nitrogenase FeMo cofactor. Science 334:940 22096190 10.1126/science.1214025 PMC3268367

[43] Dance I (2018) What is the role of the isolated small water pool near FeMo-co, the active site of nitrogenase? FEBS J 285:2972–2986. 10.1111/febs.14519 29797782 10.1111/febs.14519

[44] Groom CR, Bruno IJ, Lightfoot MP, Ward SC (2016) The Cambridge Structural Database. Acta Crystallographica Sect B-Struct Sci Crystal Eng Mater 72:171–179. 10.1107/s2052520616003954 10.1107/S2052520616003954 PMC4822653 27048719

[45] Barclay JE, Davies SC, Evans DJ, Hughes DL, Longhurst S (1999) Lattice effects in the Mossbauer spectra of salts of [Fe_4_S_4_(S(CH_2_)_*n*_OH)_4_]^2-^. Crystal structures of [PPh_4_]_2_[Fe_4_S_4_(S(CH_2_)_*n*_OH)_4_ (*n* = 2, 3 and 4). Inorg Chim Acta 291:101–108. 10.1016/s0020-1693(99)00098-5

[46] Carrell HL, Glusker JP, Job R, Bruice TC (1977) Synthetic tetranuclear iron-sulfur complex with ionized side chains. The crystal structure of (Fe_4_S_4_(S(CH_2_)_2_COO)_4_)^6-^. (N_a_5.N(_C_4_H_9_)_4^)6^+•5_C_5_H_9N*O*. J Am Chem Soc 99:3683–3690. 10.1021/ja00453a028 858874 10.1021/ja00453a028

[47] You JF, Snyder BS, Papaefthymiou GC, Holm RH (1990) On the molecular / solid-state boundary. A cyclic iron-sulfur cluster of nuclearity 18: synthesis, structure and properties. J Am Chem Soc 112:1067–1076. 10.1021/ja00159a028

[48] You JF, Snyder BS, Holm RH (1988) Na_2_Fe_18_S_30_^8-^ - a high nuclearity cyclic cluster generated solely by iron sulfur bridge bonding. J Am Chem Soc 110:6589–6591. 10.1021/ja00227a064

[49] Grunwald L, Clémancey M, Klose D, Dubois L, Gambarelli S, Jeschke G, Wörle M, Blondin G, Mougel V (2022) A complete biomimetic iron-sulfur cubane redox series. Proceed Nat Acad Sci United States Am. 10.1073/pnas.2122677119 10.1073/pnas.2122677119 PMC9351461 35881795

[50] Grunwald L, Inoue M, Carril PC, Wörle M, Mougel V (2024) Gated electron transfers at synthetic iron-sulfur cubanes. Chem. 10.1016/j.chempr.2023.09.023

[51] Johansson G, Lipscomb WN (1958) The structure of Roussin’s black salt, CsFe_4_S_3_(NO)_7_.H_2_O. Acta Crystallogr A 11:594–598. 10.1107/s0365110x58001596

[52] Berman HM, Westbrook J, Feng Z, Gilliland G, Bhat TN, Weissig H, Shindyalov IN, Bourne PE (2000) The protein data bank. Nucleic Acids Res 28:235–242. 10.1093/nar/28.1.235 10592235 10.1093/nar/28.1.235 PMC102472

[53] Adman E, Watenpaugh KD, Jensen LH (1975) NH...S hydrogen bonds in Peptococcus aerogenes ferredoxin, Clostridium pasteurianum rubredoxin, and Chromatium high potential iron protein. Proc Natl Acad Sci USA 72:4854–4858. 10.1073/pnas.72.12.4854 1061073 10.1073/pnas.72.12.4854 PMC388830

[54] Ma JC, Dougherty DA (1997) The cation-pi interaction. Chem Rev 97:1303–1324. 10.1021/cr9603744 11851453 10.1021/cr9603744

[55] Burley SK, Petsko GA (1988) Weakly polar interactions in proteins. Adv Protein Chem 39:125–189 3072867 10.1016/s0065-3233(08)60376-9

[56] Wang QR, Guo JP, Chen P (2021) The impact of alkali and alkaline earth metals on green ammonia synthesis. Chem 7:3203–3220. 10.1016/j.chempr.2021.08.021

[57] Wang JM, Liu Z, Frank J, Moore PB (2018) Identification of ions in experimental electrostatic potential maps. IUCrJ 5:375–381. 10.1107/s2052252518006292 30002838 10.1107/S2052252518006292 PMC6038950

[58] Harding MM (2002) Metal-ligand geometry relevant to proteins and in proteins: sodium and potassium. Acta Crystallographica Sect D-Biol Crystallograp 58:872–874. 10.1107/s0907444902003712 10.1107/s0907444902003712 11976508

[59] Buscagan TM, Rees DC (2023) Modeling the correlation between Z and B in an X-ray crystal structure refinement. bioRxiv. 10.1101/2023.07.04.547724 37461620 10.1101/2023.07.04.547724 PMC10350028

[60] Hoffman BM, Dean DR, Seefeldt LC (2009) Climbing nitrogenase: toward a mechanism of enzymatic nitrogen fixation. Acc Chem Res 42:609–619. 10.1021/Ar8002128 19267458 10.1021/ar8002128 PMC2684573

[61] Eberhardt WH, Crawford B, Lipscomb WN (1954) The valence structure of the boron hydrides. J Chem Phys 22:989–1001. 10.1063/1.1740320

[62] Huniar U, Ahlrichs R, Coucouvanis D (2004) Density functional theory calculations and exploration of a possible mechanism of N_2_ reduction by nitrogenase. J Am Chem Soc 126:2588–2601 14982469 10.1021/ja030541z

[63] Fay AW, Lee CC, Wiig JA, Hu YL, Ribbe MW (2011) Protocols for cofactor isolation of nitrogenase. Nitrogen Fixation: Methods Protocols 766:239–248. 10.1007/978-1-61779-194-9_16 10.1007/978-1-61779-194-9_16 21833872

[64] Bruice TC, Maskiewicz R, Job R (1975) The acid-base properties, hydrolytic mechanism, and susceptibility to O_2_ oxidation of Fe_4_S_4_(SR)_4_^–2^ clusters. Proc Natl Acad Sci USA 72:231–234 16592211 10.1073/pnas.72.1.231 PMC432277

[65] Waser V, Ward TR (2023) Aqueous stability and redox chemistry of synthetic [Fe_4_S_4_] clusters. Coordination Chem Rev. 10.1016/j.ccr.2023.215377

[66] Burns A, Watt GD, Wang ZC (1985) Salt inhibition of nitrogenase catalysis and salt effects on the separate protein-components. Biochemistry 24:3932–3936

[67] Deits TL, Howard JB (1990) Effect of salts on Azotobacter vinelandii nitrogenase activities - inhibition of iron chelation and substrate reduction. J Biol Chem 265:3859–3867 2303482

